# sRAGE alleviates SARS-CoV-2-induced pneumonia in hamster

**DOI:** 10.1038/s41392-022-00883-6

**Published:** 2022-02-02

**Authors:** Xiuqin Zhang, Dan Li, Rui Sun, Xinli Hu, Zhiqi Song, Xiaotian Ni, Hua Zhu, Tiannan Guo, Chuan Qin, Rui-Ping Xiao

**Affiliations:** 1 Institute of Molecular Medicine, College of Future Technology, Peking University, Beijing, China; 2 Key Laboratory of Human Disease Comparative Medicine of National Health Commission of the People’s Republic of China, Beijing Key Laboratory for Animal Models of Emerging and Remerging Infectious Diseases, Institute of Laboratory Animal Science, Chinese Academy of Medical Sciences and Comparative Medicine Center, Peking Union Medical College, Beijing, China; 3 Westlake Laboratory of Life Sciences and Biomedicine, Key Laboratory of Structural Biology of Zhejiang Province, School of Life Sciences, Westlake University, Hangzhou, China

**Keywords:** Translational research, Infectious diseases

**Dear Editor**

Since last year, the most demanding task of the global pharmaceutical community has been focused on the development of strategies to treat coronavirus disease 2019 (COVID-19). To date, several vaccines have been developed and already demonstrated their efficacy in reducing the incidence of COVID-19.^1^ However, the development of drugs treating COVID-19 is lagging far behind, and all the current treatment regimens have their limitations.^2^ The lung is the major and usually the initial organ to be attacked by severe acute respiratory syndrome coronavirus 2 (SARS-CoV-2). Therefore, putting lung inflammation under control is expected to alleviate whole-body inflammatory responses and inflammation-induced organ damage.

In principle, the inflammatory responses incurred by SARS-CoV-2 involve diversified upstream stimuli, multiple signaling pathways, and numerous downstream effectors.^3^ Therefore, targeting any particular cytokine or even signaling pathway may not be sufficient in alleviating the systemic overreaction of immune system in severe COVID-19, namely cytokine storm. It is urgent and pivotal to develop means which can systemically tuning down the exaggerated immune responses via a relative central node in the inflammation signaling cascades.

The receptor for advanced glycation endproducts (RAGE) is a multiligand, pro-inflammatory pattern recognition receptor that is implicated in both infectious and sterile inflammatory conditions.^4^ Upon ligand binding to the receptor, it transduces signals via several downstream kinases, including MAPKs, PI3K/Akt, and JAK, which in turn, activate transcription factors NF-κB, AP-1, and Stat3. These transcription factors promote the expression of important cytokines, such as TNF-α, IL-1, and IL-6. Remarkably, RAGE is almost exclusively expressed in the lung and involved in multiple lung diseases. Soluble RAGE (sRAGE) is a splicing variant or a post-translationally cleaved short form of RAGE which lacks the transmembrane and intracellular C-terminal domain, thus serving as a decoy receptor to attenuate inflammatory responses initiated by the full-length RAGE.^5^ However, it is unknown whether RAGE signaling plays a role in SARS-CoV-2-induced pneumonia, and if so, whether sRAGE can be applied as a therapeutic agent to treat COVID-19.

In order to investigate the role of RAGE/sRAGE in SARS-CoV-2-induced pneumonia, we used a SARS-CoV-2-inoculated hamster model of COVID-19 (Supplementary Fig. S1a) with RAGE highly expressed in the lung (Supplementary Fig. S1b). For therapeutic intervention, the infected hamsters were treated with sRAGE or human serum albumin (HSA) (Supplementary Fig. S1c), starting from day 1 post-inoculation (1dpi). SARS-CoV-2 inoculation caused severe pneumonia in the hamsters, and sRAGE treatment profoundly mitigated SARS-CoV2-induced pneumonia (Fig. 1a–c) and significantly delayed body weight loss (*F* = 2.363, *P* = 0.025) (Supplementary Fig. S1d), although the RAGE expression and the viral RNA loads in lung tissues were not different between sRAGE- and HSA-treated groups (Supplementary Fig. S1e, f). Remarkably, sRAGE significantly reduced the diffused thickened alveolar septum, multifocal exudation, and accumulation of inflammatory cells in the perivascular and peribronchial spaces in the lung (Fig. 1a, b) and sRAGE treatment also reduced the number of hamsters with severe interstitial pneumonia (Fig. 1c). Furthermore, immunohistochemical staining demonstrated that SARS-CoV-2-induced recruitment of CD3-positive T cells and the expression of myxovirus resistance protein 1 (Mx1, also known as Mx2 in hamster) in the lung were greatly reduced by sRAGE treatment (Fig. 1d), suggesting alleviated lung inflammation induced by SARS-CoV-2. Consistently, quantitative reverse transcription polymerase chain reaction (RT-qPCR) showed that the mRNA levels of the macrophage marker *CD68*, and inflammatory disease markers including *IFIT3* and *Mx1*, and inflammatory cytokines including *IL-1β*, *IL-6*, *TNF-α*, *IL-18*, and *IL-10*, as well as *ICAM1*, were induced by virus infection, while the induction was significantly repressed by the treatment with sRAGE (Fig. 1e and Supplementary Fig. S1g). These results corroborate that sRAGE can effectively suppress SARS-CoV-2-triggered pneumonia.

**Fig. 1:**
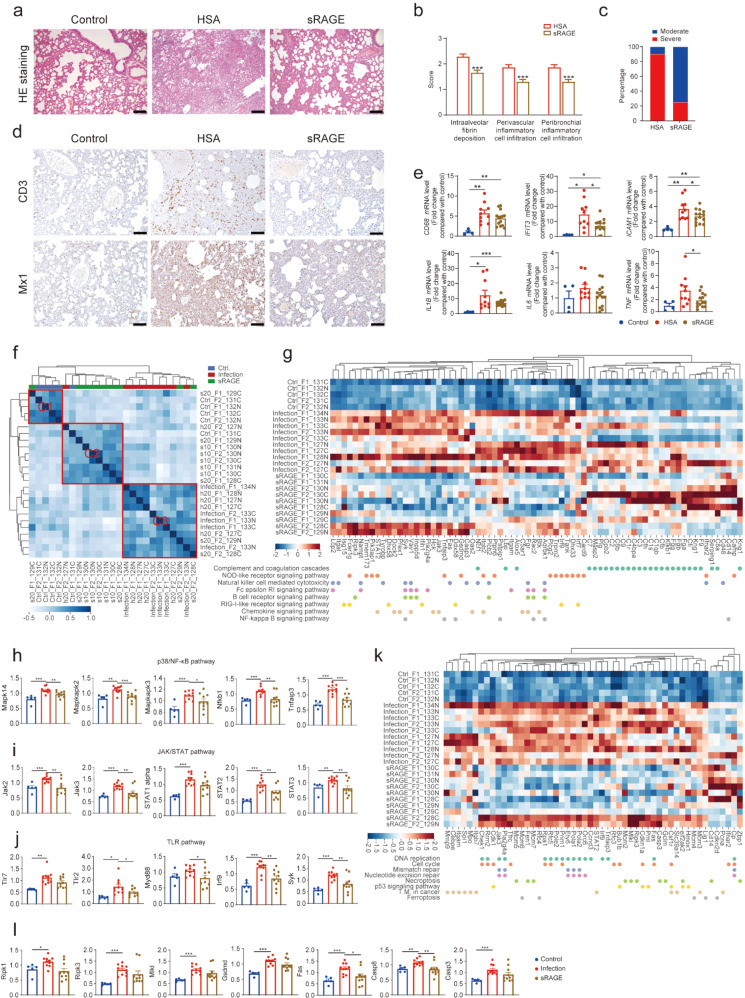
sRAGE alleviates SARS-CoV-2-induced pneumonia via inhibition of multiple signaling pathways involved in exaggerated inflammatory response and cell death. **a** Representative lung histopathological images (H&E staining) from uninfected control hamsters, HSA- or sRAGE-treated SARS-CoV-2-infected hamsters. Scale bar = 100 μm. **b** Pathological score of the lung lesions. *n* = 10 in HSA-treated group, *n* = 15 in sRAGE-treated group. **c** Percentages of severe interstitial pneumonia in HSA- or sRAGE-treated SARS-CoV-2 infected hamsters. *n* = 10 in HSA-treated group, *n* = 15 in sRAGE-treated group. **d** Immunohistochemical staining of CD3 and Mx1 expression cells in the lung. Scale bar = 100 μm. **e** Expression levels of *CD68, IFIT3, ICAM1, IL-1β, IL6, and TNF* in the lung determined by RT-qPCR. *n* = 4 in control group, *n* = 10 in infection group, *n* = 14 in sRAGE-treated group. **f** Heatmap of clustered correlation matrix. The samples were clustered into three groups: C1 (consisting 4 control, 1 infected and treated with sRAGE hamsters), C2 (consisting 7 infected and sRAGE-treated, and 1 infected and HSA-treated hamsters), and C3 (consisting 5 infected and untreated, 4 infected and treated with HSA, and 2 infected and treated with sRAGE hamsters). **g** Heatmap showing the normalized expression of inflammation-related proteins across control, infection, and sRAGE-treated groups. (**h**–**j**) Statistical analysis of protein expression in p38/NF-κB pathway (**h**), JAK/STAT pathway (**i**), and TLR pathway (**j**) in lung proteomics. *n* = 5 in control group, *n* = 10 in infection group, *n* = 10 in sRAGE-treated group. **k** Heatmap showing the normalized expression of cell cycle/death-related proteins across control, infection, and sRAGE-treated groups. **l** Statistical analysis of necroptosis, pyroptosis, and apoptosis-related protein expression in the lung proteomics. *n* = 5 in control group, *n* = 10 in infection group, *n* = 10 in sRAGE-treated group. Data are mean ± SEM. *P < 0.05, **P < 0.01, ***P < 0.001 (Student’s *t*-test)

To systematically analyze the molecular pathogenesis in the lung upon SARS-CoV-2 infection and sRAGE treatment, we used stable isotope-labeled proteomics analysis (TMTpro, 16plex) to profile the whole proteome of the lung tissues. The list of the proteins identified and analyzed are shown in Supplementary data S1. The hierarchical clustering analysis separated the sRAGE-treated samples from those untreated or treated with HSA (Fig. 1f). Thus, in the following analysis, the samples from untreated or HSA-treated groups were combined into one group (labeled as “infection”), and the samples from sRAGE-treated animals were labeled as “sRAGE”. Consistently, principal component analysis (PCA) also separated the samples into three groups based upon their proteomic profiles (Supplementary Fig. S2a). Importantly, sRAGE treatment attenuated the increases of 74.7% (408 out of 546) of the SARS-CoV-2 infection-upregulated proteins (Supplementary Fig. S2b and Supplementary data S1—sheet 4). Several major pathways affected by virus infection were identified using Reactome or KEGG analysis of the 546 upregulated proteins (Supplementary Fig. S2c, d and Supplementary data S1—sheet 5, 6). Most of the upregulated proteins involved in inflammation and DNA replication upon SARS-CoV-2 infection were downregulated by sRAGE treatment according to the recovery score (Supplementary Fig. S2e, f).

The infection of SARS-CoV-2 instigated profound inflammatory responses in the lung, as evidenced by the upregulation of multiple inflammation-related proteins, while most of these changes were ameliorated by sRAGE treatment (Fig. 1g). Immunohistochemical staining in lung tissues revealed that the sRAGE treatment resulted in the downregulation of total and phosphorylated p65 transcription factors and their nuclear localization (Supplementary Fig. S3a), as well as reduced signal intensity of total and phosphorylated MAPK p38 proteins in the sRAGE treated lungs (Supplementary Fig. S3b). In line with these observations, proteomic data confirmed that the infection-induced alterations in the NF-κB and p38 signaling were repressed by sRAGE (Fig. 1h). Furthermore, the upregulation of JAK/STAT signaling components caused by SARS-CoV-2 infection was also mitigated by sRAGE treatment (Fig. 1i). In addition to these known downstreams of RAGE, the Toll-like receptor signaling cascades were also curbed by sRAGE treatment as all the protein levels of TLR7, TLR2, Myd88, IRF9, and Syk tended to decline in response to sRAGE treatment (Fig. 1j). Moreover, other elevated proteins related to inflammatory signaling, including Cdk7, Ddx58, Dock2, and Ifih, were also restored by sRAGE treatment (Supplementary Fig. S3c). Taken together, the above results strongly indicate that treatment with sRAGE suppresses the virus-triggered, exaggerated inflammatory responses of multiple inflammatory signaling pathways.

Importantly, SARS-CoV-2 infection-induced activation of cell cycle/death-related pathways that was also alleviated by sRAGE treatment (Fig. 1k). Particularly, several key factors involved in inflammatory cell death, such as necroptosis-related Ripk1, Ripk3, and Mlkl, pyroptosis-related Gsdmd, as well as apoptosis-related Fas, Caspases 8 and 3, were all decreased in response to sRAGE treatment (Fig. 1l), which should contribute to the reduced cell death in the lung (Supplementary Fig. S4a, b). In fact, TUNEL positive cells were greatly reduced not only in the lung but also in the heart and kidney (Supplementary Fig. S4a, b), regardless of the absence of obvious histological changes in both heart and kidney tissues (Supplementary Fig. S4c), suggesting that sRAGE treatment ameliorates the systemic tissue damage caused by SARS-CoV-2 infection.

In summary, we have performed the first “proof-of-concept” study of using sRAGE to treat COVID-19 in the hamster model. The results have demonstrated that sRAGE can potently and systemically attenuate the overactivation of inflammatory responses triggered by SARS-CoV-2 infection. A combination of sRAGE with certain anti-viral drugs may provide a more effective treatment for COVID-19. Our study provides strong evidence supporting the therapeutic potential of using sRAGE in the real clinical settings.

## Supplementary information

Supplementary information-clean

Dataset 1

## Data Availability

All the datasets used and/or analyzed during this study are available from the corresponding author on reasonable request.
