# Supplementary material for: sRAGE alleviates SARS-CoV-2-induced pneumonia in hamster
Source: Signal Transduct Target Ther. 2022 Feb 2;7:36. doi: 10.1038/s41392-022-00883-6 (PMC8809059; doi:10.1038/s41392-022-00883-6)
Supplement: Supplementary file 1 — Supplementary information-clean [file 41392_2022_883_MOESM1_ESM.pdf]

Supplementary Materials for

sRAGE alleviates SARS-CoV-2-induced pneumonia in hamster

Xiuqin Zhang[1#], Dan Li[2#], Rui Sun[3#], Xinli Hu[1#], Zhiqi Song[2#], Xiaotian Ni[1], Hua Zhu[2], Tiannan Guo[3*], Chuan Qin[2*], Rui-Ping Xiao[1*]
Correspondence to: Rui-Ping Xiao (xiaor@pku.edu.cn, Chuan Qin (qinchuan@pumc.edu.cn); or Tiannan Guo (guotiannan@westlake.edu.cn)

This PDF file includes:

Materials and Methods
Figures. S1 to S4
Tables S1

Materials and methods

SARS-CoV-2 virus

The SARS-CoV-2 virus (accession number is MT093631.2, SARS-CoV-2/WH-09/human/2020/CHN) [1] was used in this study. Experiments associated with the virus were conducted in a biosafety level 3 (ABSL3) facility using HEPA-filtered isolators.

Experimental animal

Pathogen-free, 8-10 weeks old, male, and female Syrian hamsters were obtained from BEIJING HFK BIOSCIENCE. Animal studies were performed in an animal biosafety level 3 (ABSL3) facility using HEPA-filtered isolators. All procedures in this study involving animals were reviewed and approved by the Institutional Animal Care and Use Committee of the Institute of Laboratory Animal Science, Peking Union Medical College (QYJ20007).

In vivo animal study

A total of 30 male and female hamsters were used in this study. The experimental procedure was similar to the previous study [1]. Briefly, the hamsters were divided to infection group and non-infection group. The hamsters in the infection group were inoculated intranasally with the SARS-CoV-2 stock virus with a dosage of 10^6 50% tissue culture infections dose ($TCID_{50}$) in 200 µl PBS; the hamsters in non-infection group were inoculated intranasally with 200µl PBS. In the infected group, human recombinant sRAGE (at the dose of 20 ng/g and 10 ng/g) (Sino Biological Inc., Beijing, China; 11629-HCCH). was intraperitoneally injected at 1, 2, 3, 4, 5, and 6 dpi as treatment, and human serum albumin (HSA, 20 ng/g) (HEOWNS biocom-technology LLC., Tianjin, China; R-70020) was used as treatment control; the purity of sRAGE and HSA were tested by Coomassie blue staining (Supplementary Fig. S1c). All the hamsters were observed daily to record body weights and clinical symptoms. The hamsters were sacrificed at 7 dpi, the lungs were collected for viral load analyses, pathological examinations, and mechanistic studies. Other organs were also

collected for histological examinations.

Viral load detection

Viral load was determined by RT-qPCR. Whole lung homogenates were prepared using an electric homogenizer, and the total RNA was extracted from the homogenate by using the RNeasy Mini Kit (Qiagen, Hilden, Germany). The reverse transcription was performed using the PrimerScript RT Reagent Kit (TaKaRa, Japan) according to the manufacturer's instructions. RT-qPCR and viral load calculation were performed according to the previous study [2].

Proteomics

Formalin-fixed paraffin-embedded (FFPE) samples were analyzed using TMT-based proteomics as described previously [3,4] In brief, after paraffin of FFPE samples were removed by heptane (Sigma-Aldrich, Germany), the formalin-fixed samples were rehydrated and reversed cross-links with acid, base, and heat. Next, the proteins were denatured with 6 M urea (Sigma-Aldrich, Germany) and 2 M thiourea (Sigma-Aldrich, Germany), followed by PCT-assisted digestion into peptides with trypsin (Hualishi, Beijing, China) at a 1:20 enzyme-to-substrate ratio and Lys-C (Hualishi, Beijing, China) at a 1:80 enzyme-to-substrate ratio.

Cleaned peptides were labeled with TMTpro™ 16 plex (Thermo Fisher Scientific™, San Jose, USA) [5]. The TMT126 channel was to label a pooled peptide sample, while the other 15 channels were used for 15 samples to be analyzed. The 30 concatenated fractions per batch were separated using an offline high-pH reverse phase with a Thermo Scientific™ UltiMate™ 3000 RSLC System.

Subsequently, the fractionated sample was separated with Thermo Scientific™ UltiMate™ 3000 RSLCnano System. The mass spectrometer was operated in positive mode with the FAIMS Pro interface and then analyzed with an Orbitrap Exploris 480 mass spectrometry by data-dependent acquisition (DDA) mode (Thermo Fisher Scientific™, San Jose, USA). The optimal compensation voltage (CV) was set to -48V and -68V with a cycle time of 1 s per FAIMS experiment. MS1

resolution was set at 60,000 with a normalized AGC target 300%. Mass range was set to 375-1800. Dynamic exclusion mode was set to custom with an exclusion duration of 40 s. The MS2 resolution was set to 30,000 with a normalized AGC target of 200%. Isolation window was 0.7 *m/z* and first mass was set to 100 *m/z*. Normalized HCD collision energy was 36%. Turbo-TMT was enabled and MS/MS data was recorded in centroid mode. The database searching included all *Mesocricetus auratus* entries from UniProt (20 May 2021, containing 31,983 proteins) using Proteome Discoverer (version 2.4, Thermo Fisher Scientific™, Waltham, MA) as described previously [6,7].

Bioinformatic analysis of proteomics data

The correlation analysis and hierarchical clustering were carried out by scipy (version 1.5.4) package with python. The principal component analysis was carried out by scikit-learn (version 0.24.2) package with python. Protein differential excretion was assessed with the Kruskal-Wallis test. Pathway enrichment analysis was performed using KEGG and Reactome.

The recovery score was defined as below:

$$Recovery\ score\ = -\ log_2\, Ratio(sRAGE, Infection)$$

Where $Ratio(sRAGE, infection)$ is defined as:

$$Ratio(sRAGE, infection)\ =\ \frac{abundance(sRAGE)}{abundance(infection)}$$

Western blot

Lung tissues were lysed with RIPA lysis buffer (Solarbio, R0010) supplemented with protease inhibitor and phosphatase inhibitors on ice for 30min. Tissue lysates were then centrifuged at 12,000 rmp/min at 4°C for 15 min, and then the supernatants were collected and inactivated for the virus at 56°C for 30 min, above experiments were performed in a Biosafety Level 3 facility. 30 μg proteins were subjected to western blot analysis, and an anti-RAGE (Abcam, ab216329) or anti-β-actin (Yeasen, 30101) antibody was used to detect the protein expression.

RT-qPCR

To analyze the mRNA expression in the lung, whole lung homogenates were prepared by using an electric homogenizer, and total RNA was extracted from the homogenate by using the RNeasy Mini Kit (Qiagen). cDNA was prepared with cDNA Synthesis SuperMix (TransGen, AE311-04) according to the manufacturer's protocols. The gene expression was analyzed using SuperReal PreMix Plus SYBR Green (TIANGEN BIOTECH, FP205). Actb was used as the reference gene. Data were analyzed with the ΔCt method. Primers were designed based on the predicted sequences of *Mesocricetus auratus* in the NCBI database (Table S1).

Pathological examination

The Hamsters were sacrificed and autopsies were performed at 7dpi. The lungs and other organs were grossly examined and then fixed in 10% buffered formalin solution, and paraffin sections (3-4 μm in thickness) were prepared. H&E staining and immunohistochemistry were used to identify histopathological changes in the lungs. The H&E stained sections were diagnosed by two pathologists who were blinded to the experimental groups, and semiquantitative pathology scoring was conducted based on alveolar wall thickening, intra-alveolar fibrin deposition (exudation), and inflammatory cell infiltration. Mild (+), the lesion area is less than 1 / 4 of the lung section; moderate (++), the lesion area is about 1 / 4 ~ 2 / 4 of the lung section; severe (+++), the lesion area is about 2 / 4 ~ 3 / 4 of the lung section; severest (++++), the lesion area is more than 3 / 4 of the lung section.

Immunohistochemistry

For immunohistochemical staining, paraffin sections were incubated with anti-CD3 (ZSGB-BIO, ZM-0417), anti-Iba1 (Wako, 019-19741), anti-MxA (EMD Millipore, MABF938), anti-p38 (Cell Signaling, #8690), Phospho-p38 (Cell Signaling #4631), NF-κB p65 (Cell Signaling, #8242) or Phospho-NF-κB p65 (ThermoFisher, 44-711G) antibody overnight at 4°C followed by incubation

with goat anti-rabbit or goat anti-mouse horseradish peroxidase-labeled secondary antibody (Dako Diagnostics) for 1 hr at room temperature. After being counterstained with hematoxylin, sections were observed under a light microscope (BX53, Olympus, Tokyo, Japan) and images were obtained by cellSens Standard software (Olympus, Tokyo, Japan).

TUNEL staining

To detect cell death in situ, TUNEL (terminal deoxynucleotidyl transferase dUTP nick-end labeling) staining was performed in paraffin-embedded sections using a One-Step TUNEL Assay Kit (Beyotime, C1090) following the manufacturer's instructions. The stained sections were imaged under a fluorescence microscope (IX71, Olympus), and the $TUNEL^+$ cells in 200× images were counted. The number of $TUNEL^+$ cells in each animal was the average number from 5 ~ 6 of 200× images.

Statistical analysis

Statistical analysis was performed with Prism software (GraphPad Software). Data are presented as means ± SEM. A two-way ANOVA with Dunnett's multiple comparisons test was used to compare differences between the sRAGE-treated group and the HSA-treated group for the body weight loss. And two-tailed Student's *t*-test was used to compare differences between control and treated groups or between different treated groups for other parameters. P values ≤ 0.05 was considered statistically significantly different. *, $p < 0.05$; **, $p < 0.01$; ***, $p < 0.001$.

References

1 Deng, W. *et al.* Ocular conjunctival inoculation of SARS-CoV-2 can cause mild COVID-19 in rhesus macaques. *Nat Commun*. **11**, 4400, (2020).

2 Song, Z. *et al.* SARS-CoV-2 Causes a Systemically Multiple Organs Damages and Dissemination in Hamsters. *Front Microbiol*. **11**, 618891, (2020).

3 Zhu, Y. *et al.* High-throughput proteomic analysis of FFPE tissue samples facilitates tumor stratification. *Mol Oncol*. **13**, 2305-2328, (2019).

4 Gao, H. *et al.* Accelerated Lysis and Proteolytic Digestion of Biopsy-Level Fresh-Frozen and FFPE Tissue Samples Using Pressure Cycling Technology. *J Proteome Res*. **19**, 1982-1990, (2020).

5 Li, J. *et al.* TMTpro reagents: a set of isobaric labeling mass tags enables simultaneous proteome-wide measurements across 16 samples. *Nat Methods*. **17**, 399-404, (2020).

6 Shen, B. *et al.* Proteomic and Metabolomic Characterization of COVID-19 Patient Sera. *Cell*. **182**, 59-72 e15, (2020).
7 Nie, X. *et al.* Multi-organ proteomic landscape of COVID-19 autopsies. *Cell*. **184**, 775-791 e714, (2021).

Fig. S1

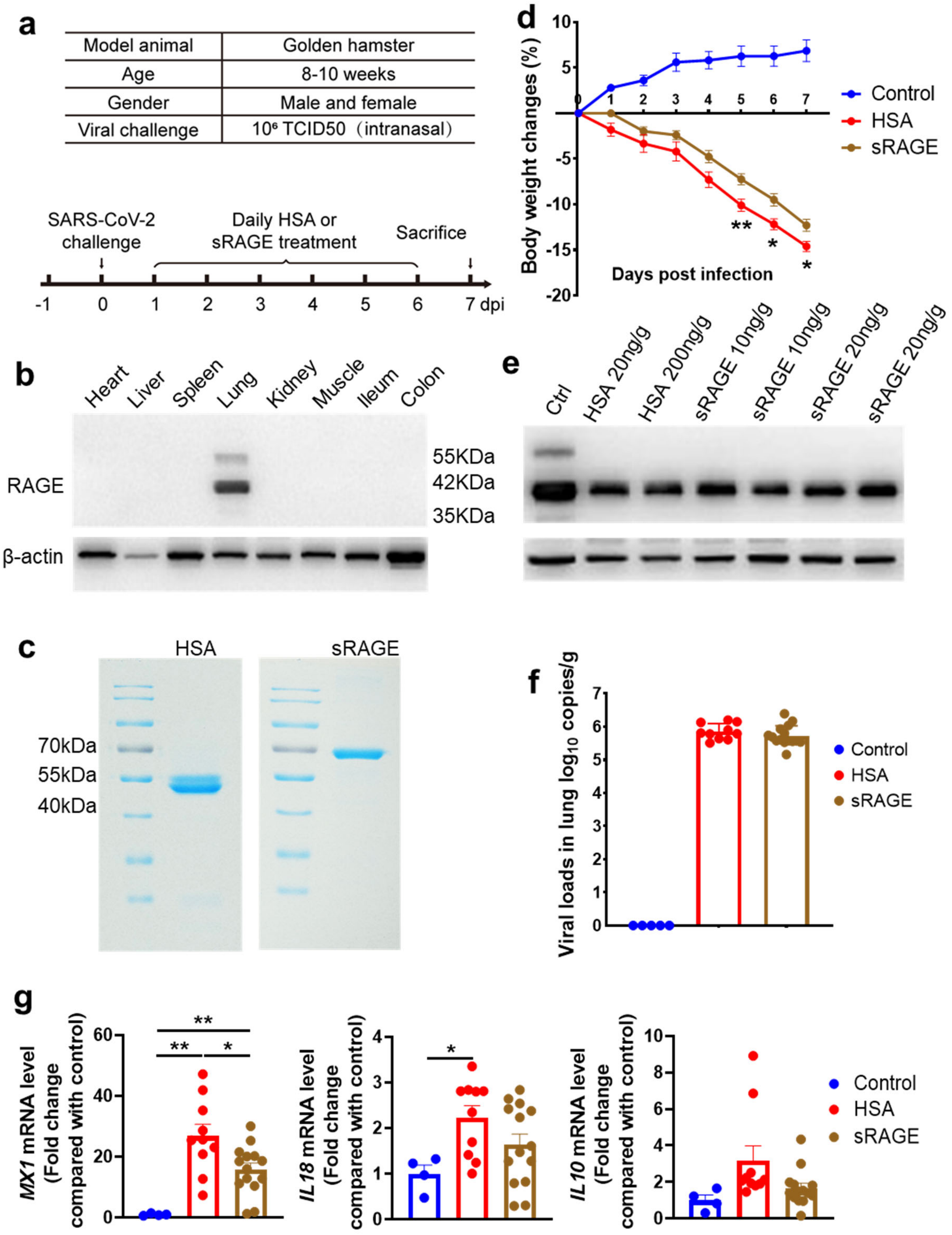
a
Model animal
Golden hamster
Age
8-10 weeks
Gender
Male and female
Viral challenge
10⁶ TCID50 (intranasal)
SARS-CoV-2 challenge
Daily HSA or sRAGE treatment
Sacrifice
-1 0 1 2 3 4 5 6 7 dpi
b
Heart Liver Spleen Lung Kidney Muscle Ileum Colon
RAGE
55KDa
42KDa
35KDa
β-actin
c
HSA
sRAGE
70kDa
55kDa
40kDa
d
Body weight changes (%)
Days post infection
Control
HSA
sRAGE
e
Ctrl HSA 20ng/g HSA 200ng/g sRAGE 10ng/g sRAGE 10ng/g sRAGE 20ng/g sRAGE 20ng/g
f
Viral loads in lung log10 copies/g
Control
HSA
sRAGE
g
MX1 mRNA level (Fold change compared with control)
IL18 mRNA level (Fold change compared with control)
IL10 mRNA level (Fold change compared with control)
Control
HSA
sRAGE

Fig. S1 sRAGE reduced SARS-CoV-2-induced body weight loss and inflammatory cytokine expression in the lung. **a** Study design of hamster model of COVID-19. **b** Western blot showing RAGE expression in hamster. **c** Coomassie blue staining of sRAGE (2μg) and HSA (2μg). **d** Body weight changes in control, sRAGE or HSA-treated SARS-CoV-2 infected hamsters. n = 5 in control group, n = 15 in sRAGE-treated group, n = 10 in HSA-treated group. Data are mean ± SEM. *P<0.05, **P<0.01, (two-way ANOVA with Dunnett's multiple comparisons test). **e** Western blot showing RAGE expression in control, sRAGE- or HSA-treated SARS-CoV-2-infected hamster lungs. **f** Viral load in the hamster lung at 7 dpi. **g** RT-qPCR detection of *Mx1*, *IL-18* and *IL-10* expression in the lung tissue. n = 4 in control group, n = 10 in infection group, n = 14 in sRAGE-treated group. Data are mean ± SEM. *P<0.05, **P<0.01, (Student's *t*-test)**.**

Fig. S2.

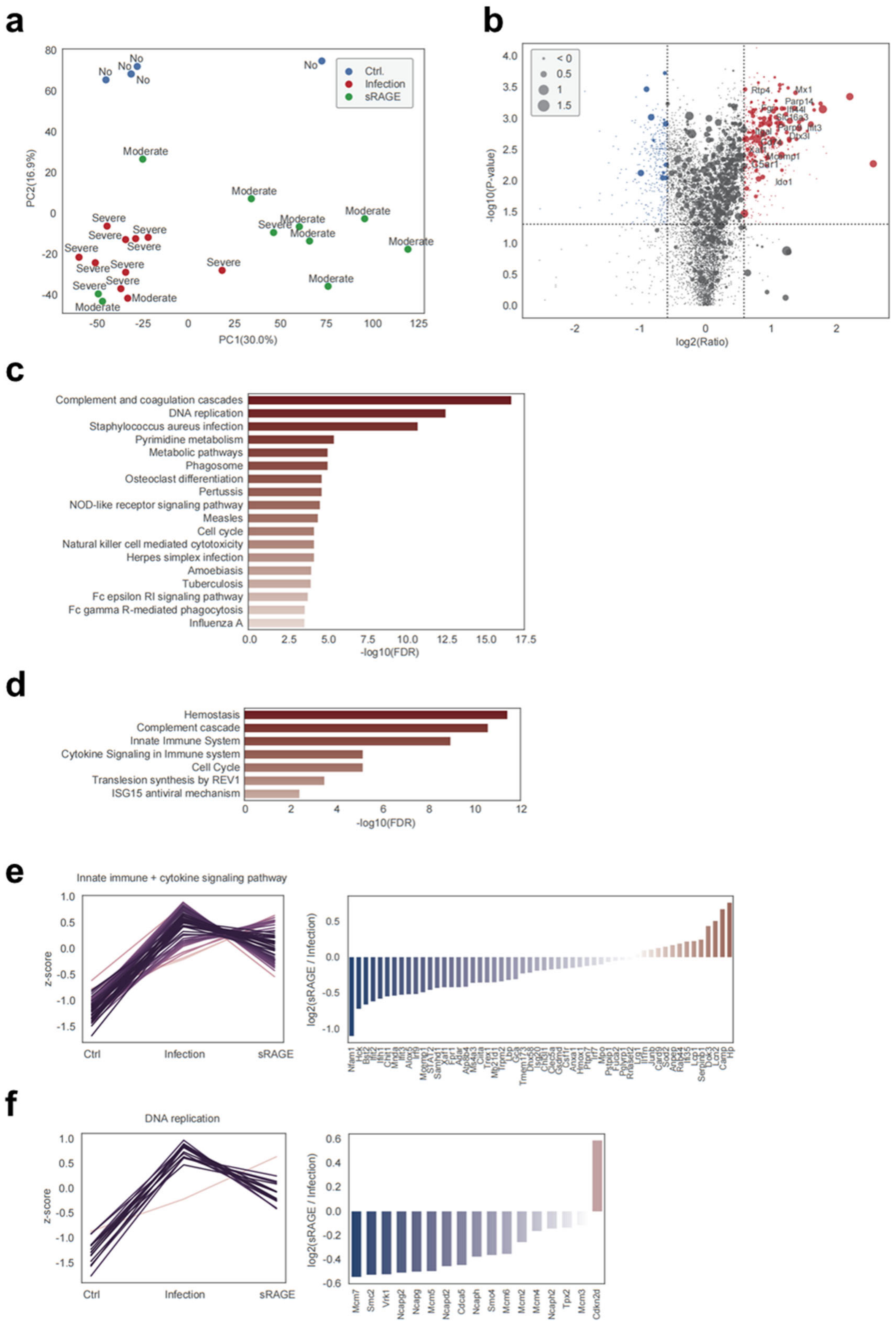
a
PC1(30.0%)
PC2(16.9%)
Ctrl.
Infection
sRAGE
No
Moderate
Severe
b
log2(Ratio)
-log10(P-value)
< 0
0.5
1
1.5
Rtp4
Mx1
Parp14
Ido1
c
Complement and coagulation cascades
DNA replication
Staphylococcus aureus infection
Pyrimidine metabolism
Metabolic pathways
Phagosome
Osteoclast differentiation
Pertussis
NOD-like receptor signaling pathway
Measles
Cell cycle
Natural killer cell mediated cytotoxicity
Herpes simplex infection
Amoebiasis
Tuberculosis
Fc epsilon RI signaling pathway
Fc gamma R-mediated phagocytosis
Influenza A
-log10(FDR)
d
Hemostasis
Complement cascade
Innate Immune System
Cytokine Signaling in Immune system
Cell Cycle
Translesion synthesis by REV1
ISG15 antiviral mechanism
-log10(FDR)
e
Innate immune + cytokine signaling pathway
z-score
Ctrl
Infection
sRAGE
log2(sRAGE / Infection)
f
DNA replication
z-score
Ctrl
Infection
sRAGE
log2(sRAGE / Infection)
Mcm7
Smc2
Vrk1
Ncapg2
Ncapg
Mcm5
Ncapd2
Cdca5
Ncaph
Smc4
Mcm6
Mcm2
Mcm4
Ncaph2
Tpx2
Mcm3
Cdkn2d

Fig. S2 Analysis of protein profiles in hamster lungs. a Principal component analysis of the samples based on protein profiles. **b** Volcano plot displaying the differentially expressed proteins between the controls and the infections. The x-axis corresponds to the log2-transformed ratio of the mean abundance of infections to controls. The y-axis corresponds to the -log10 of the Kruskal p-value between controls and infections. The size of the dot corresponds to the recovery score (i.e. the degree of attenuation after sRAGE treatment). The larger the size of the dot, the greater the degree of attenuation. **c** Enrichment of the KEGG pathways of the infection-upregulated proteins. **d** Enrichment of the Reactome pathways of the infection-upregulated proteins. **e** The left panel represents the trends of the proteins related to innate immune system and cytokine signaling pathway after infection and treatment. The right panel represents the abundance ratio (sRAGE-treated to infection) of these proteins. **f** The left panel represents the trends of the proteins related to DNA replication after infection and treatment. The right panel represents the abundance ratio (sRAGE-treated to infection) of these proteins.

Fig. S3.

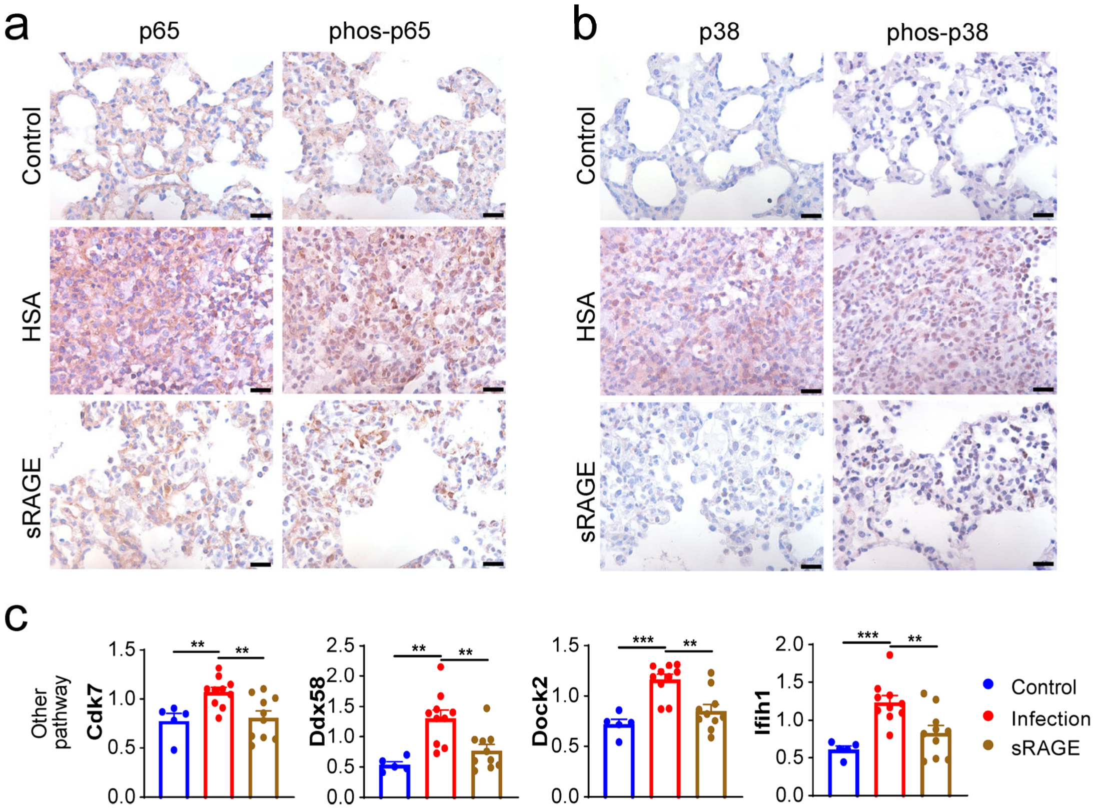

Fig. S3 sRAGE attenuated SARS-CoV-2-induced multiple inflammatory responses. a Immunohistochemical staining showing p65 and phosphorylated-p65 expression and distribution in the lung. Scale bar = 20 μm. **b** Immunohistochemistry showing p38 and phosphorylated-p38 expression in the lung. Scale bar = 20 μm. **c** Statistical analysis of protein expression in inflammatory related proteins in lung proteomics. n = 5 in control group, n = 10 in infection group, n = 10 in sRAGE-treated group. Data are mean ± SEM. ***P*<0.01, ****P*<0.001 (Student's *t*-test).

Fig. S4.

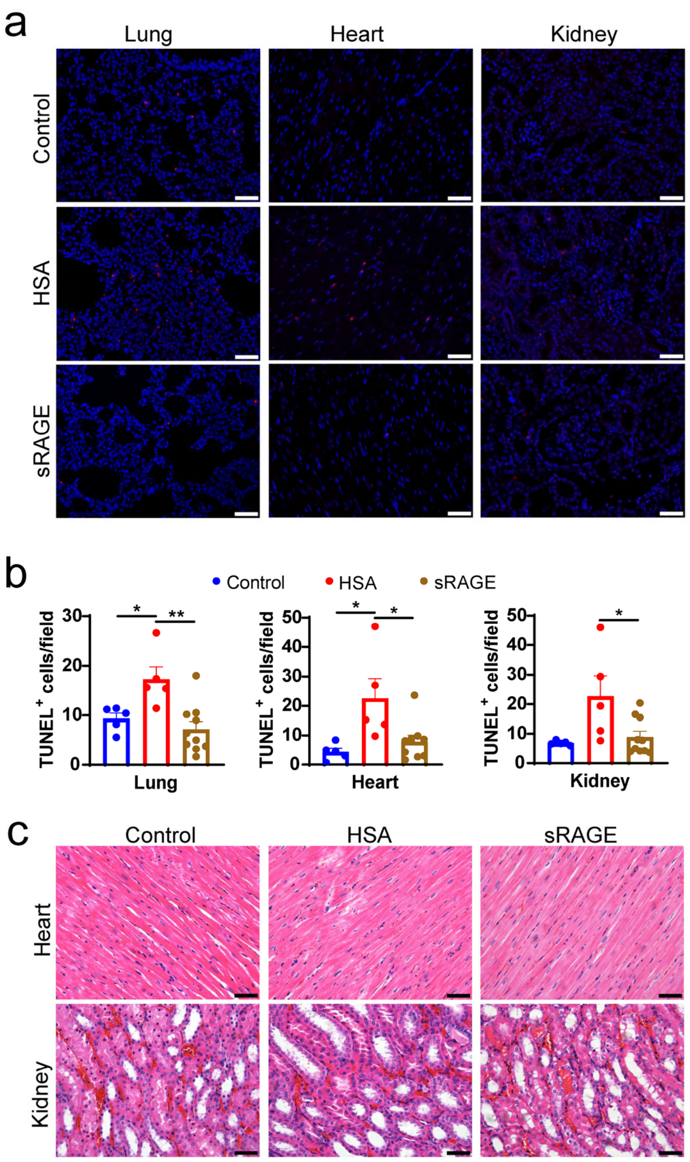

Fig. S4 sRAGE treatment ameliorated cell death in multiple organs. a TUNEL staining of the sections of the lung, heart, and kidney. Scale bar = 50 μm. **b** The statistics results of TUNEL staining of the lung, heart and kidney sections. n = 5 in control group, n = 5 in HSA-treated group, n = 10 in sRAGE-treated group. **c** Heart and kidney histopathological images (H&E staining) from control, sRAGE, or HSA-treated SARS-CoV-2 infected hamsters. Scale bar = 50 μm. Data are mean ± SEM. **P*<0.05, ***P*<0.01, (Student's *t*-test).

Table S1 Primer sequences for RT-qPCR

Gene	Forward	Reverse
MX1	GGTATCGTTACCAGGTGCCC	GGTCTGGAACACTTGGGGAG
IL10	TTACTGGCTGGAGTGGGACC	GTCCAGCTGGTCCTTCTTTTGAA
IFIT3	GAACCGTACAGTCCACACCC	GGTGAACTCACTCATGATGGAAA
IL1B	GCAGTTCCCCAACTGGTACA	TAAATCCTGGCCGCTGTTGT
IL6	GGAGTGGCCAAGAACCAAGA	ATGCTAAGGCACAGCACACT
TNF	AGAATCCGGGCAGGTCTACT	TATCCCGGCAGCTTGTGTTT
IL18	TGCACACTGGATTGAAGTGGT	GTGGATCCAGCAGCAGTCAT
CD68	GCCTGGGGCATCGCTATATT	AGACAGGTGGGGATAGGCAT
ICAM1	AGGCTGTGGCAAGACAAGAT	CATGTCACAGAAACACGCCC
ACTB	*AGAAGCTGTGCTATGTTGCC*	*CCACAGGATTCCATACCCAGG*
